# Supplementary material for: Analysis of the RNA-Dependent RNA Polymerase 1 (RDR1) Gene Family in Melon
Source: Plants (Basel). 2022 Jul 7;11(14):1795. doi: 10.3390/plants11141795 (PMC9320487; doi:10.3390/plants11141795)
Supplement: Supplementary file 1 [file plants-11-01795-s001.zip › plants-1748399-supplementary.pdf]

plants

Table S1. Melon genotypes tested for RDR1b mapping.

No	Name	Origin, source
1	FLT47	Var flexuosus, Italy
2	Cha-T	Var cantaloupensis, Charentais T, France
3	Top Mark	Var cantaloupensis (reticulatus), USA
4	Ein Dor	Var cantaloupensis, Ananas type, Israel
5	MEA	Var Inodorus, Melona Amarilla, Spain
6	MR-1	Muskmelon [52]
7	Pl 161375	Var conomon, SON, Korea
8	CHT	Var chito, Pl 164320, India
9	ZM3	Var agrestis, TGR 228, Zimbabwe
10	CIN	Var conomon, Pl 420176, Ginsen Makuwa, Japan

Table S2. List of viruses.

Virus	Genus	Accession No	Reference
ZYMV-Is	Potyvirus	EF062582	[53]
CMV Fny-strain	Cucumovirus	D10538	[54]
CMV-Δ2b	Cucumovirus		[36]
CGMMV	Tobamovirus	KF155232	[55]
CVYV	Ipomovirus	AY290865	[56]
WMV	Potyvirus	AF322376	[57]
PRSV-W	Potyvirus	AED88251	[58]

Table S3. List of RDR genes used. *CmRDR1c1* and *CmRDR1c2* were Sanger sequenced for this study, using primers obtained from the sequences of the cucumber analogues and the Melonomics database. All other genes were accessed from public databases (accessed in 28 February 2020). * Gene numbers from Figure 3

Cucumis melo	Accession number	Database
RDR1a (13)*<br>rdr1b1 (43)<br>rdr1b2 (43)<br>RDR1c1 (14)<br>RDR1c2 (15)	KT316427<br>melo3c026813t1<br>melo3c026814t1<br>MW192784<br>MW192785	(http://www.ncbi.nlm.nih.gov/pubmed)<br>(https://www.melonomics.net)<br>(https://www.melonomics.net)<br>(http://www.ncbi.nlm.nih.gov/pubmed)<br>(http://www.ncbi.nlm.nih.gov/pubmed)
RDR2 16)	melo3c017106t1	(https://www.melonomics.net)
RDR6 (17)	melo3c011257t1	(https://www.melonomics.net)
Cucumis sativus		
RDR1a (1)	KT316424	(http://www.ncbi.nlm.nih.gov/pubmed)
RDR1b (2)	KT316425	(http://www.ncbi.nlm.nih.gov/pubmed)
RDR1c1 (3)	KT316426	(http://www.ncbi.nlm.nih.gov/pubmed)
RDR1c2 (4)	KX442651	(http://www.ncbi.nlm.nih.gov/pubmed)
RDR2 (5)	Csa1M005580.1	(http://www.icugi.org)
RDR6 (6)	Csa2M381730.1	
Citrullus lanatus		(http://www.icugi.org)
RDR1a (7)	Cla001937	
RDR1b (8)	Cla002214	
RDR1c1 (9)<br>RDR1c2 (10)	Cla003960<br>Cla003946	
RDR2 (11)	Cla019179	
RDR6 (12)	Cla022323	
Cucurbita pepo		(http://www.icugi.org)
RDR1a (18)	Cp4.1LG01g05850.1	
RDR1b (19)	Cp4.1LG01g05940.1	
RDR1c1 (20)	Cp4.ILG01g18460.1	
RDR1c2 (21)	Cp4.ILG13g06620.1	
RDR2 (22)	Cp4.1LG18g08320.1	
RDR6 (23)	Cp4.1LG11g03220.1	
Cucurbita moschata		(http://www.icugi.org)
RDR1a (24)	CmoCh04C000890.1	
RDR1b (25)	CmoCh04C000870.1	
RDR1c1 (26)<br>RDR1c2 (27)	CmoCh04C022260.1<br>CmoCh15G009380.1	
RDR2 (28)	CmoCh10G001440.1	
RDR6 (29)	CmoCh05G003850.1	
Solanum tuberosum		(https://solgenomics.net)
RDR1a (38)	Sotub05g010500.1.1	
RDR1b (39)	Sotub05g010490.1.1	
Solanum lycopersicum		(https://solgenomics.net)
RDR1 (30)	Solyc05g007510.2.1	
Nicotiana tabacum		(http://www.ncbi.nlm.nih.gov/pubmed)
RDR1 (35)	AJ011576	
Hordeum vulgare		(http://www.ncbi.nlm.nih.gov/pubmed)
RDR1a (36)	ACH53360	
RDR1b (37)	ACH53361	
Gossypium hirsutum		(http://www.ncbi.nlm.nih.gov/pubmed)
RDR1 (34)	NP_001314013.1	
Brachypodium distachyon		(http://phytozome.jgi.doe.gov/pz/portal.html)
RDR1 (31)	Bradi3g59910	
RDR2 (32)	Bradi1g33920	
RDR6 (33)	Bradi1g55350	
Arabidopsis thaliana		(http://www.ncbi.nlm.nih.gov/pubmed)
RDR1 (40)	NP_172932	
RDR2 (41)	NP_192851	
RDR6 (42)	NP_190519	

Table S4. Primers used in this study

Gene	Purpose	Primers Forward (F) Reverse (R)	Primer sequence (5'-end to 3'-end)
CmRDR1a	PCR/Q-PCR	(F)<br>(R)	gatccattgaaggctcagga<br>gatacggctcgacatctggt
Cmrdr1b1	PCR	(F)<br>(R)	ctaagagatggcatcaccattg<br>cttctcatccacacgactgc
Cmrdr1b2	PCR	(F)<br>(R)	tgccaatgctcatacagctt<br>tttcggaaagtcaactgcaa
Cmrrdr1b<br>*CsRDR1b*	PCR<br>(unknown sequence verification)	(F)<br>(R)	ctaagagatggcatcaccattg<br>tttcggaaagtcaactgcaa
CmRDR1c1/c2	PCR	(F)<br>(R)	ctgtccctggaagatcaagc<br>tcaatagcatcgagcaaacg
CmRDR1c1	Q-PCR	(F)<br>(R)	tccattgctgttgacttttcg<br>tcaaaggagttgacatccgaa
CmRDR1c2	Q-PCR	(F)<br>(R)	tccattgctgttgacttccca<br>tcaaaggagttgacatccgaa
CmRDR2	PCR/Q-PCR	(F)<br>(R)	tccattgctgttgacttccca<br>tcaaaggtgttgacatcagat
CmRDR6	PCR/Q-PCR	(F)<br>(R)	taccgccgaattagagatgc<br>cctgccacttcgaggtctac
Cyclophilin AY942800	PCR/Q-PCR	(F)<br>(R)	gttgctttaaggactccgcca<br>aggggtacctccgcatagctag
**Viruses**			
ZYMV CP	Q-PCR	CP-489 (F)<br>CP-639 (R)	gccgcgtctttcgaagataa<br>gaaccaagaggcgaattgct
CMV-Fny<br>CMV-Δ2b	Q-PCR	RNA3 (F)<br>RNA3 (R)	ctgatctgggcgacaagga<br>cgataacgacagcaaaacac
CGMMV	Q-PCR	CP (F)<br>CP (R)	gtttcgcttctcagctccac<br>cgcgtcatcagtacgcttta
CVYV CP	Q-PCR	CP (F)<br>CP (R)	attcccaagctcagcaaaga<br>cgaacctttctatctcccaatg
PRSV-W	Q-PCR	CP (F)<br>CP (R)	gaatggtacatcaccggacata<br>cggagtggcatgctctatta
WMV	Q-PCR	CP (F)<br>CP (R)	aacgatgagcaaatgggtgt<br>gtttgggtaatgatggacgg
**Construct design**			
Mel-RDR1c-sgRNA-SalI (BmgBI)	Construction of pRCS-35S:Cas9-AtU6:RDR1c-sgRNA	(F)	3'-agagtcgacatagcgattgagagaatg gaagacgtgaagttttagagctagaaatag ca-5'
U6-sgRNA HindIII	Construction of pRCS-35S:Cas9-AtU6:RDR1c-sgRNA	(R)	gctaagcttagaactaaaaaaagcac
**Genotyping and mutant verification**			
Cas9	Transgene verification	(F)<br>(R)	gacactgacggctttatgcc<br>gctaagcttcgatctaaaaaaagcac
RDR1c1	PCR/Restriction analysis for mutation detection; sequencing	(F)	tttatttacttgattgtccg
RDR1c2	PCR/Restriction analysis for mutation detection; sequencing	(F)	gtaatttacttaattatcca
RDR1c1/c2	PCR/Restriction analysis for mutation detection; sequencing	(R)	cttggaaggttgattgagagg

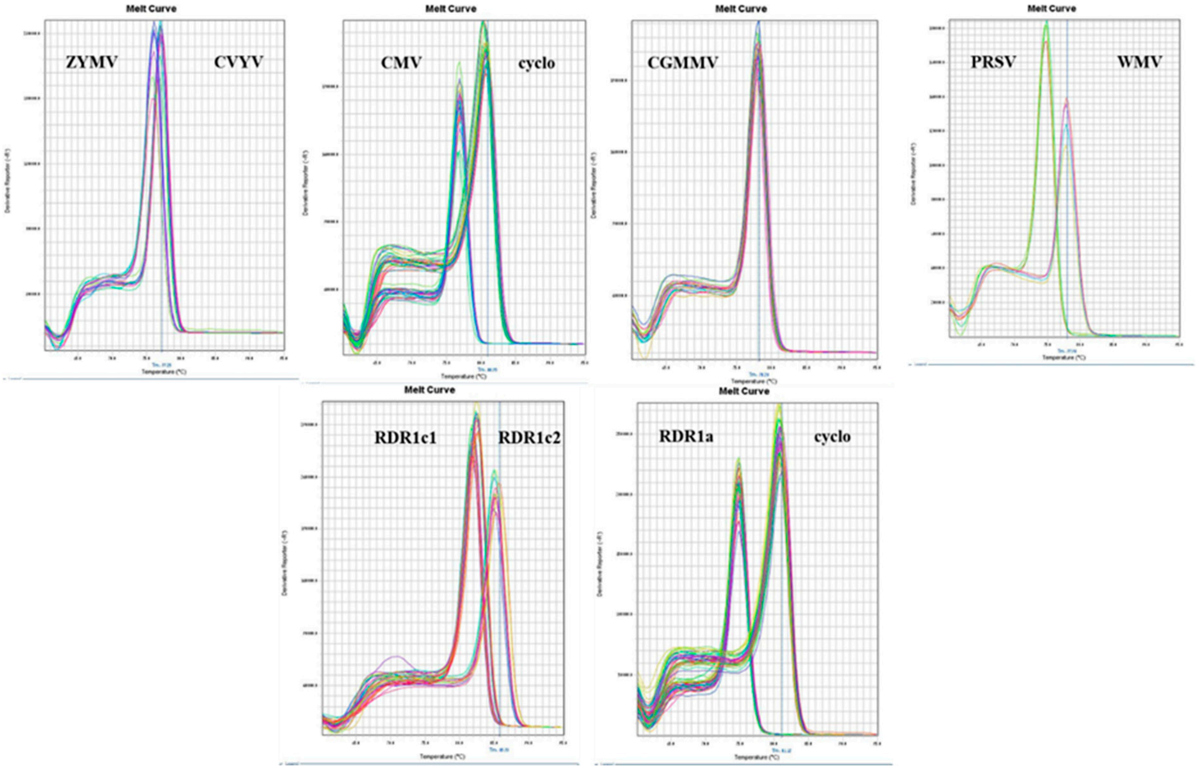

Figure S1. Melting curves analysis of RT-PCR **with specific primers to viruses and *CmRDRs* in melon**

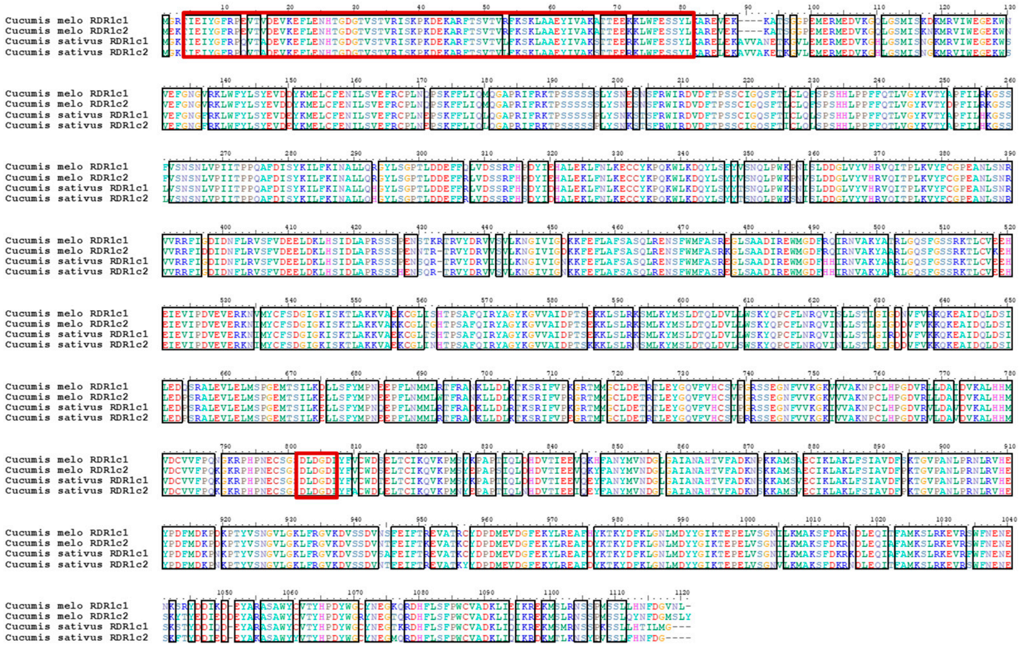

Figure S2. Amino acid sequence alignment of putative proteins encoded by the *CmRDR1c1*, *CmRDR1c2*, *CsRDR1c1* and *CsRDR1c2* genes of melon cv. Arava and cucumber cv. Beit-Alfa. Black boxes indicate identical amino acids. Red-labelled boxes indicate the RNA binding domain (4-82) and the RDR1 five-amino-acid conserved catalytic domain (802-806). Accession numbers of RDR genes are listed in Table S3.

REFERENCES FOR SUPPLEMENTARY MATTER

36. Kumari, R.; Kumar, S.; Leibman, D.; Abebie, B.; Shnaider, Y.; Ding, S.-W.; Gal-On, A. Cucumber RDR1s and cucumber mosaic virus suppressor protein 2b association directs host defence in cucumber plants. *Mol. Plant Pathol.* **2021**, *22*, 1317–1331. https://doi.org/10.1111/mpp.13112.
52. Wechter, W.P.; Whitehead, M.P.; Thomas, C.E.; Dean, R.A. Identification of a randomly amplified polymorphic DNA marker linked to the Fom 2 Fusarium wilt resistance gene in muskmelon MR-1. *Phytopathology* **1995**, *85*, 1245–1249.
53. Gal-On, A. A Point Mutation in the FRNK Motif of the Potyvirus Helper Component-Protease Gene Alters Symptom Expression in Cucurbits and Elicits Protection Against the Severe Homologous Virus. *Phytopathology* **2000**, *90*, 467–473. https://doi.org/10.1094/PHYTO.2000.90.5.467.
54. Rizzo, T.M.; Palukaitis, P. Construction of full-length cDNA clones of cucumber mosaic virus RNAs 1, 2 and 3: Generation of infectious RNA transcripts. *Mol. Gen. Genet. MGG* **1990**, *222*, 249–256. https://doi.org/10.1007/BF00633825.
55. Reingold, V.; Lachman, O.; Blaosov, E.; Dombrovsky, A. Seed disinfection treatments do not sufficiently eliminate the infectivity of Cucumber green mottle mosaic virus (CGMMV) on cucurbit seeds. *Plant Pathol.* **2015**, *64*, 245–255. https://doi.org/10.1111/ppa.12260.
56. Martínez-garcía, B.; Marco, C.F.; Goytia, E.; López-abella, D.; Serra, M.T.; Aranda, M.A.; López-moya, J.J. Development and use of detection methods specific for Cucumber vein yellowing virus (CVYV). *Eur. J. Plant Pathol.* **2004**, *110*, 811–821. https://doi.org/10.1007/s10658-004-2491-7.
57. Desbiez, C.; Lecoq, H. The nucleotide sequence of Watermelon mosaic virus (WMV, Potyvirus) reveals interspecific recombination between two related potyviruses in the 5′ part of the genome. *Arch. Virol.* **2004**, *149*, 1619–1632. https://doi.org/10.1007/s00705-004-0340-9.
58. Ali, A. First Complete Genome Sequence of Papaya ringspot virus-W Isolated from a Gourd in the United States. *Genome Announc.* **2017**, *5*, 1434–1516. https://doi.org/10.1128/genomeA.01434–16.
